# Clinical Differences of mild, Moderate, and Severe Gambling Disorder in a Sample of Treatment Seeking Pathological Gamblers in Sweden

**DOI:** 10.1007/s10899-022-10183-x

**Published:** 2023-01-07

**Authors:** Mikael Mide, Elin Arvidson, Anna Söderpalm Gordh

**Affiliations:** 1 Department of Addiction and Dependency, Sahlgrenska University Hospital, Journalvägen 5, 416 50 Gothenburg, Sweden; 2 Addiction Biology Unit, Department of Psychiatry and Neurochemistry, Institution of Neuroscience and Physiology, The Sahlgrenska Academy at University of Gothenburg, Gothenburg, Sweden

**Keywords:** *Comorbidity*, *DSM-5*, *Gambling*, *Gambling Disorder*, *Severity*, *Treatment-seeking*

## Abstract

**Introduction:** Gambling disorder (GD) is classified among the addictive disorders in the DSM-5 and the severity of the diagnosis can be specified as mild, moderate and severe. It has been seen that individuals with more severe gambling problems have a higher rate of comorbid disorders and other health problems compared to individuals with a milder clinical picture. **Aims:** The aim of this study was to explore clinical psychiatric differences related to the severity of disorder in treatment-seeking patients with GD. **Method:** A sample of 163 patients with GD seeking treatment at an outpatient clinic was diagnosed using the SCI-GD, screened for comorbid diagnoses using the MINI, and further completed a range of self-report questionnaires measuring alcohol-, and drug-problems, symptoms of depression and anxiety, emotion regulation, cognitive distortions, and quality of life. **Results:** Greater severity was associated to more problems with alcohol and illicit drugs. Severe gamblers were more likely to gamble to “escape”, and had more symptoms of depression and anxiety. Participants with moderate and severe gambling disorder had more difficulties with emotion regulation. Cognitive distortions were the same between severities. All groups had Quality-of-Life problems at a clinical level. **Discussion:** There are some distinctive differences between GD of different severities. The features shown by patients with severe GD indicates a more emotionally vulnerable group with increased symptom severity. Further knowledge about the features of GD severity levels is important for treatment planning in the clinic.

## Introduction

In Sweden and in most places worldwide there has been a rapid increase in the chances to gamble for money through substantial marketing and easy online access. Today about 1.3% of the Swedish population is estimated to have some degree of gambling problems and 0.6% have severe gambling problems (Public Health Agency of Sweden, 2019). Worldwide the prevalence of problem gambling is estimated at between, 0.5–7.6%, with Sweden’s prevalence rates being in the average range (Williams et al., 2012). When specifically assessing active online gamblers, studies have reported a prevalence of problem gambling ranging between 2.7% to as high as 57.2% (Mora-Salgueiro et al., 2021). Prevalence studies have also found that pathological gambling is associated with a low degree of treatment seeking, with only about 7 − 10% with a lifetime prevalence of pathological gambling having ever accessed healthcare services for their gambling problems (Cunningham, 2005; Slutske, 2006).

Since 2013, GD is the only behavioral addiction recognized and listed in the Diagnostic and Statistical Manual Version 5 (DSM-5) in the category of Substance-Related and Addictive Disorders (APA, 2013). GD was reclassified from an impulsivity disorder to a substance use disorder due to similarities regarding symptoms, genetic liability, biological dysfunctions, and treatment approach with other substance use disorders (Hasin et al., 2013). In addition, just as in substance use disorders, severity is classified by a simple symptom count as a higher symptom count should represent a higher likelihood of risk factors and negative consequences (Hasin et al., 2013). Now, GD is defined as a persistent gambling behavior manifested by four or more out of the following nine criteria during the past year: (1) the need to gamble for increasing amounts of money (2) restlessness or irritability when attempting to cut down or stop gambling (3) repeated unsuccessful attempts to cut down, control or stop gambling (4) preoccupation with gambling (5) often gambles when feeling distressed (6) returns to “get even” after losing money gambling (chasing losses) (7) lies to conceal the extent of involvement in gambling (8) has jeopardized or lost significant relationship, job, educational or career opportunity because of gambling and (9) relies on others to provide money to relieve desperate financial situations caused by gambling. Severity of the disorder is specified by counting the number of criteria fulfilled, with 4–5 criteria classified as mild, 6–7 as moderate and 8–9 as severe GD (APA, 2013).

Problem and pathological gambling is associated with high rates of comorbidities both in population studies and among treatment-seeking gamblers. In a meta-analysis of population representative samples, it was seen that both problem and pathological gamblers show high rates of psychiatric comorbidities (Lorrains et al., 2011). It was found that on average 37.9% of pathological and problem gamblers have a comorbid mood disorder, 37.4% a comorbid anxiety disorder, and 28.8% have an antisocial personality disorder. Substance use disorders were also common, with on average 60.1% having a nicotine dependency, 28.1% an alcohol use disorder and 17.2% an illicit drug use disorder. Further, in meta-analyses of treatment-seeking problem and pathological gamblers it was found that on average 23.1% had a comorbid mood disorder, 17.6% a comorbid anxiety disorder, 56.4% a nicotine dependency, 21.1% an alcohol use disorder, 7.0% an illicit drug use disorder, 9.3% an attention-deficit hyperactivity disorder and 47.9% any personality disorder (Dowling et al., 2015a, b).

In community studies, there also seems to be an association between the severity of gambling problems and the rate of psychiatric comorbidities and other clinically relevant factors. In a study of 31,830 adults categorized in three levels of gambling severity, “no gambling or low frequency gambling”, “low-risk or at-risk gambling”, and “problem or pathological gambling” it was found that severity was associated with the frequency of mood disorders, anxiety disorders, substance use disorders and personality disorders with for the most part a more severe gambling problem indicating a higher prevalence of disorder (Barry et al., 2011). Similarly, another study assessed the association between gambling severity and personality disorders in a sample of 13,543 adults from the U.S. and found a general trend of increased prevalence of personality disorders from non-gamblers through at-risk and problem gamblers, to pathological gamblers (Ronzitti et al., 2018). Further, in a study of 42,038 adults categorized in five gambling severity groups (non-gambling, low-risk, at-risk, problem gambling, and pathological gambling) it was found that more severe gambling problems was associated with a number of behavioral problems of which several where of an antisocial character (i.e. stealing, scamming for money). The majority of these associations remained even when controlling for antisocial personality disorder (Moghaddan et al., 2015). Furthermore, in a study of 2303 adults categorized as non-problem, low severity, and moderate/high severity gamblers it was found that moderate/high severity gamblers had higher odds of poor diet, low physical exercise, and poor general health than non-problem gamblers. Both low and moderate/high severity were associated with an increased rate of tobacco use. Low severity was associated with higher frequency of risky alcohol consumption. More severe gambling was also associated with lower mental wellbeing (Butler et al., 2020). These community studies encompass a broad range of individuals. Gamblers have been categorized into groups ranging from non-gamblers to pathological gamblers using either self-report questionnaires (Butler et al., 2020) or clinical interviews (Barry et al., 2011; Moghaddan et al., 2015; Ronzitti et al., 2018). However, in the clinic, it is pathological gamblers that present themselves for treatment. Thus, it is important to understand how gambling severity of diagnosed pathological gamblers influence the clinical picture.

Regarding treatment-seeking gamblers, the picture is less clear. It has been found in a meta-analysis on comorbid disorders that when categorizing studies according to severity (problem gamblers vs. pathological gamblers), the problem gamblers had a lower estimate of alcohol problems than pathological gamblers. No differences were found regarding depression. For other comorbidities, there were not enough studies to make comparisons (Dowling et al., 2015a). Likewise, another meta-analysis on treatment-seeking gamblers found no difference regarding prevalence of personality disorders between different gambling severities (Dowling et al., 2015b). In a Spanish study from 2001 it was found that in 69 pathological gamblers seeking treatment, a presence of more than one comorbid psychiatric disorder such as for example alcohol dependency, antisocial personality disorder, and mood disorders, was associated with greater severity of gambling (Ibáñez et al., 2001). More, in a study of 237 treatment seeking pathological gamblers in the US it was found that fulfilling criteria for an antisocial personality disorder was associated with higher severity of gambling problems (Pietrzak & Petry 2005). Also, in a study of 149 treatment seeking pathological gamblers in the US it was found that severity of gambling was associated with childhood maltreatment (Petry et al., 2005). To sum up, the literature on clinical features associated with the severity of diagnosed pathological gamblers and treatment-seeking gamblers is relatively scarce, and the findings are mixed. Also, in these studies the severity of the disorder has rarely been categorized using the diagnostic criteria of the DSM-5.

To our knowledge, there are only two such studies that use the DSM-5 criteria to categorize severity into mild, moderate, and severe GD. The first one examined clinical differences between the three severity groups in a sample of 574 individuals with GD. It was found that individuals with moderate and severe GD had an earlier gambling debut, lost more money due to gambling, consumed more nicotine, scored higher on measures of anxiety and depressive symptoms, and had lower quality of life. There were no differences between the moderate and severe group on these measures. Additionally, there were no differences between any of the groups regarding categorical mood, anxiety or substance use disorders (Grant et al., 2017). The other study explored the predictive capacity of the severity levels on relapse and drop-out during treatment with Cognitive Behavioral Therapy in 398 males with GD. They found that the severity of GD did not predict rate of drop-out nor relapse during treatment (Mestre-Bach et al., 2019).

In our study, we aimed to further investigate the clinical features of patients with GD representing the different severity levels from the DSM-5. However, clinical features presented by gamblers in the clinic could also vary due to other patient characteristics than gambling disorder severity. It has been found that non-substance related comorbid psychiatric disorders are more common in women than men among treatment seeking pathological gamblers (Håkansson et al., 2018). Nationally representative studies have also shown that major depression is more common among women in general around the world (Salk et al., 2017). Comorbid psychiatric disorders are also more common among treatment seeking problem gamblers with Attention Deficit Hyperactivity Disorder (ADHD) (Brandt & Fischer, 2019). It is also known that ADHD in general often presents with other psychiatric comorbidities (Banaschewski et al., 2017). A higher rate of cognitive distortions related to gambling has been found in pathological gamblers with ADHD (Romo et al., 2016) and of older age (Stojnić et al., 2019). Finally, associations between problems with emotion regulation has been found with ADHD (Mestre-Bach et al., 2021) as well as with older age and male gender (Sancho et al., 2019) among pathological gamblers. Thus, when exploring possible differences in clinical features between pathological gamblers of different severity levels, it is important to control for possible confounders.

Therefore, the aim of this study was to investigate clinical differences seen between treatment-seeking patients diagnosed with low, moderate and severe gambling disorder while controlling for possible confounders (gender, age and possible ADHD). We hypothesize that there are clinically relevant differences between the different severities of GD.

## Method

### Study Design

This was a descriptive study, where data was collected from individuals seeking voluntary treatment at the Clinic for Gambling Addiction and Screen Health between May 2019 and May 2021. We collected demographic variables and assessed the degree of gambling severity and prevalence of other psychiatric diagnoses. Furthermore, we assessed other clinically relevant outcomes such as additional addictive behaviours, quality of life and gambling related cognitive distortions among these individuals. The information was obtained from a number of semi-structured interviews and standardized questionnaires. No power calculation was carried out to determine the sample size. The study was approved by the Swedish Ethical Review Authority, dnr 764 − 18, and was conducted according to the 1964 declaration of Helsinki.

### Participants

The participants (n = 165) were recruited from the Clinic of Gambling and Screen Health at Sahlgrenska University hospital in Gothenburg, Sweden, the largest public health outpatient facility offering treatment for pathological gambling, in the region Västra Götaland in Sweden. Västra Götaland is a region with 1.6 million inhabitants and the clinic is located in Gothenburg with approximately 1 million inhabitants. The clinic welcome gambling disordered patients from 18 years of age and the treatment is based on cognitive behavioural therapy. Patients were referred to the clinic either by self-referral or by referral from a physician or other healthcare professional. No specific inclusion- or exclusion criteria were set. All patients that attended their first assessment at the clinic was asked about participation in the study. As participants were able to decline, this can be seen as a non-probaility convenience sampling method.

### Procedure

All patients at the clinic underwent an initial assessment consisting of a semi-structured anamnestic interview and a semi-structured diagnostic interview for diagnosing GD. In addition to demographic data collection, several questionnaires were also administered, measuring e.g., various aspects of mental health and quality of life. Some of the questionnaires has been exchanged over time due to clinical considerations and is therefore available only for a limited number of participants. Patients also underwent a psychiatric structured diagnostic interview. Due to clinical considerations this was only done for patients that did not have another psychiatric contact outside the clinic. At their first visit at the clinic, the patients were informed about the study and approved participation by signing an informed consent (IC) form in connection to the visit.

### Measures

#### Clinical Interviews

*Structured Clinical Interview for Gambling Disorder (SCI-GD)* is a semi structured guide for interviewing patients with suspected GD. It is based on the diagnostic criteria for GD in the latest version of the DSM-5 (APA, 2013). If four or more of the criteria were met the patient was diagnosed with GD. Fulfilling four to five criteria counts as mild GD, six to seven as moderate GD and eight to nine as severe GD. The SCI-GD was validated in a sample of 72 participants being screened for a treatment study. The instrument has high inter-rater reliability (kappa = 1.00) and excellent test re-test reliability on the number of GD criteria endorsed (r = .97) (Grant et al., 2004).

*The Mini- International Neuropsychiatric Interview (M.I.N.I*.) is a brief structured interview based on diagnostic criteria in DSM-5 and ICD-10. Specific questions are asked by the therapist and the patient answers all questions with “yes” or “no”. The instrument was validated in a sample of 636 psychiatric patients and controls and showed high concordance with similar instruments. It showed excellent inter-rater reliability (all kappa values over 0.75) and very good test re-test reliability (61% of kappa values over 0.75) (Sheehan et al., 1998). The M.I.N.I. has also been studied in a Swedish context and demonstrated good acceptability in a clinical setting (Pettersson et al., 2018) and is recommended by Swedish health authorities for use in addictive care (Socialstyrelsen, 2019).

*Anamnestic interview.* In the anamnestic interview questions were asked about tobacco use, drug use and other psychiatric diagnoses besides gambling. Information related to gambling was also collected; age of gambling debut, how many years since gambling became a problem, the function of gambling (e.g., economic reasons) and dominant gambling type (e.g., sports betting). This anamnestic interview was created on site for use in the specific clinical setting and has not been validated.

#### Self-report Questionnaires

*The NORC Diagnostic Screen for Gambling Problems (NODS)* (latest 30 days) is a self-report questionnaire measuring severity of gambling problems based on the diagnostic criteria in DSM-5. The instrument consists of 17 questions with response alternatives “yes” or “no”. Severity of gambling problems is classified into three categories based on the number of questions answered by “yes”: risk gambling, problem gambling and pathological gambling (Hodgins, 2004). The NODS was validated in a sample of 157 male military veterans and was shown to have adequate construct validity. It was also found to have good internal consistency (α = 0.88) and excellent test-retest reliability (r = .99) (Wickwire et al., 2008).

*Patient Health Questionnaire (PHQ-9)* consists of nine items screening for symptoms of depression during the last two weeks. PHQ-9 was developed according to diagnostic criteria in the DSM-IV and the total score can be used to assess severity of depressive symptoms. Based on the total score the level of severity is classified as none (0–4), mild (5–9), moderate (10–14), moderately severe (15–19) or severe (20–27). The PHQ-9 was validated in a sample of 3000 patients from a primary care setting and another 3000 patients from an obstetrics-gynecology setting, and showed good construct validity, excellent internal validity (α = 0.89 in the primary care setting and α = 0.86 in the obstretics-gynecology setting) and excellent test-restest reliability (r = .84) (Kroenke et al., 2001).

*Generalised Anxiety Disorder Assessment (GAD-7*) was developed as an instrument to measure presence and grade symptoms of anxiety. It is a seven-item questionnaire screening for symptoms during the last two weeks. The total score is 21, with cut off points at 5, 10 and 15, indicating minimal (0–4), mild (5–9), moderate (10–14) and severe (15–21) levels of anxiety. It was validated in a sample of 2149 primary care patients, and showed good construct validity, excellent internal consistency (α = 0.92) and good test-retest reliability (r = .83) (Spitzer et al., 2006).

*Gambler´s beliefs questionnaire (GBQ)* is a measure of cognitive distortions related to gambling. The questionnaire consists of 20 statements regarding thoughts connected to gambling. The participant responds by indicating to what degree the statements are correct on a seven-point Likert scale ranging from “strongly agree” to “strongly disagree”. Higher scores indicate more irrational cognitions related to gambling (Steenbergh et al., 2002). The GBQ has been validated in a Swedish context, in a sample of 402 participants recruited from the general population and from populations with former and current gambling problems. It was demonstrated to have good construct validity and excellent internal consistency (α = 0.94) (Mide et al., 2022). Test-retest reliability was evaluated in the original study and was found to be adequate (r = .77) (Steenbergh et al., 2002). Pathological gamblers tend to show more cognitive distortions than non-pathological gamblers on the GBQ (Winfree et al., 2013).

*Alcohol Use Disorders Identification Test (AUDIT)* screens for alcohol related problems. It consists of 10 items divided into three areas: alcohol consumption, symptoms of dependence and negative consequences of alcohol consumption. It has been psychometrically evaluated in a sample of 997 participants from a general population in Sweden. A cut-off score of 6 for women and 8 for men was found to indicate hazardous or harmful drinking, with the total score being 40 points. It has good internal consistency (α = 0.82) and excellent test-retest reliability (r = .93) (Bergman & Källmén, 2002).

*Drug Use Disorders Identification Test (DUDIT)* is screening for use of illicit drugs and events of drug-related consequences. It is an 11-item instrument with a maximum score of 40. The questions are categorized in the three areas drug use, dependence symptoms and negative consequences of drug use. In a sample of 1500 participants from the general population, it was found that DUDIT scores of 1 or more for women and 3 or more for men indicated problematic drug use. The DUDIT has good internal consistency (α = 0.80) (Berman et al., 2005).

*The World Health Organisation adult ADHD self-report scale (ASRS-V1.1)* is identifying adult individuals with symptoms of ADHD. The eighteen items are based on symptoms described in DSM-IV with a five-point response scale ranging from “never” to “very often”. It was psychometrically evaluated in a sample of 154 respondents from a US national comorbidity survey. The first 6 items, were found to have better classification accuracy than the full scale, with 97.9% (Kessler et al., 2005). It has good internal consistency (α = 0.89) and test-retest reliability (r = .88) (Kim et al., 2013).

*Brunnsviken Brief Quality of life scale (BBQ)* measures an individual´s subjective quality of life in a clinical setting. It is divided into six different life areas such as “view on life”, “creativity” and “friends and friendship”. Total score is 96 with higher scores indicating higher perceived quality of life. The BBQ was evaluated in a sample of n = 163 undergraduate students and a clinical sample of n = 568 participants seeking treatment for social anxiety disorder. Evidence suggested it to have good construct validity. It has adequate internal consistency (α = 0.76) and good test-retest reliability (r = .82) (Lindner et al., 2016).

*Difficulties in Emotion Regulation Scale (DERS-16)* is measuring difficulties in the regulation of emotions. It consists of 16 statements concerning reactions to emotional discomfort. Response alternatives is graded from 1 (almost never) to 5 (almost always). The scoring ranges from 16 to 80, with higher scores representing larger difficulties in emotion regulation. DERS-16 is divided in the five subscales clarity, goals, impulse, strategies and non-acceptance. The DERS-16 was validated in three different samples, n = 96 enrolled in a group-therapy treating deliberate self-harm, n = 102 from the general population, and n = 482 from a multi-site study of emotion dysregulation. It was demonstrated to have good construct validity in all samples. It also has excellent internal consistency (α = 0.92) and good test-retest reliability (ρ_I_ = 0.85) (Bjureberg et al., 2016).

*Demographic data questionnaire* acquires a number of demographic aspects from the participants including age, sex, educational level, civil status, housing situation and current occupation. This demographic questionnaire was created for the study and has not been previously examined in other studies.

### Missing data

During the length of the study 225 patients with gambling problems attended a first visit at the clinic. Informed consent was received from a total of 165 patients during the length of the study, leaving 27% (n = 60) patients that declined to participate. Criteria for GD on the SCI-GD (≥ 4 symptoms) were not met for n = 2 participants. These were excluded. This left a total of n = 163 participants included in the analysis.

Due to changes in the measurement battery over time (based on clinical considerations) ASRS was only administered to n = 45 participants. Also, based on clinical considerations the MINI was only administered to n = 124 participants. In some cases, questionnaires had been left unanswered by participants. These were treated as missing. Frequency of missing data for these questionnaires was between 4 and 9%.

### Data Analysis

The majority of analyses were run in IBM SPSS Statistics version 25. ANCOVAS and subsequent post hoc tests were run in SAS version 9.4. The statistical analysis plan was created in consultation with a statistician. Severity of Gambling Disorder (GD) was categorized based on the three levels indicated in the DSM-5 (APA, 2013): mild, moderate, and severe. The results from the SCI-GD were used for this categorization. A descriptive analysis of histograms of the non-categorical variables showed that most of these were normally distributed or only slightly skewed in a way that parametric statistics was deemed viable. The exceptions were: duration of gambling problems, AUDIT and DUDIT which were all severely skewed. Participants were asked about their dominant gambling type/s and reason/s for gambling in the semi-structured anamnestic interview. Participants could answer one or several gambling types and reasons. The answers were clustered into five categories for gambling type: online slots/casino, sports betting (online and offline), gambling in a physical store (bingo, slots, poker, horses), day trading and Other. Reasons for gambling were clustered into five categories: financial, escape, excitement, habit, and self-harm. As participants could answer with several gambling types and reasons, each participant could end up in several categories. The frequency of each category was reported in %. AUDIT and DUDIT were reported both as mean scores with standard deviations, and also as frequency of harmful alcohol use and problematic drug use. AUDIT scores ≥ 6 for women and ≥ 8 for men indicate potential harmful alcohol consumption (Bergman & Källmén, 2002). DUDIT scores ≥ 1 for women and ≥ 3 for men indicate problematic drug use (Berman et al., 2005).

In order to assess if there were any differences between participants with different severities of GD in the ordered demographic variable education level, and in nominal variables with only two categories (tobacco use, each gambling type, and each reason for gambling), the Mantel-Haenszel test for trend was used (Mantel, 1963). Regarding education level, gambling type: physical store, gambling type, daytrading, and reason: self-harm, the exact test was used as > 20% of cells had expected counts < 5. For nominal demographic variables with more than two categories (occupational status, living situation) Fisher’s exact test (Fisher, 1922) was used as > 20% of cells had expected counts < 5. When significant differences were found, odds ratios were calculated.

Due to gambling severity being an ordered variable, it was possible to use the Jonckheere-Terpstra test for trend (Jonckheere, 1954) to test if there were statistically significant trends for age, age of gambling debut, duration of gambling problems, alcohol problems (AUDIT), illicit drug problems (DUDIT) and NODS score with increasing GD severity. The Jonckheere-Terpstra test was used as it is more powerful than a Kruskal-Wallis test when comparing medians, and because it also tests a hypothesized ordering of the groups (Ali et al., 2015). Mann-Whitney U tests (Mann & Whitney, 1947) was used for pairwise comparisons. To assess possible differences between different severities of GD for all dependent measures included in the main analysis (PHQ-9, GAD-7, DERS-16, BBQ and GBQ-SE), and at the same time control for confounders, Analysis of covariance (ANCOVA) was used. The variables that on a theoretical basis was decided to be possible confounders and thus included as covariates were: gender, age, and possible ADHD (Håkansson et al., 2018; Banaschewski et al., 2017; Mestre-Bach et al., 2021; Sancho et al., 2019; Salk et al., 2017; Romo et al., 2016; Stojnić et al., 2019; Brandt & Fischer, 2019). Possible ADHD was defined as either having an ADHD diagnosis in the medical records, or positively screening for ADHD on the MINI and/or ASRS. The Tukey-Kramer test was used for pairwise comparisons. For significant differences found in pairwise comparisons, a 95% confidence interval for mean difference was calculated.

## Results

### Sociodemographic Characteristics

In Table 1, frequencies, means and standard deviations of demographic variables, alcohol and drug use in the total sample and categorized by gambling severity are presented. Participants (n = 163) in this study were categorized into the three different severities of GD indicated in the DSM-5 (American Psychiatric Association, 2013) based on results from the SCI-GD. Fulfilling 4–5 criteria were considered a mild GD (n = 22), 6–7 criteria a moderate GD (64) and 8–9 criteria a severe GD (n = 77). Most of the participants were men, 74.8%, and most, 80.9%, had at least a high-school degree. A total of 72% had a day job, whereas the remaining 28% were otherwise occupied, with sick leave being the most common, 14.9%. Living alone, 30.6%, was most common followed by living with a partner and children, 25.6%. The average age of participants was 35.2 years.

**Table 1:** *Demographics of total sample, mild, moderately and severely disordered gamblers. Data are presented as means and standard deviations M (SD) and in percent (%)*

	Total sample (n = 163)	Mild GD (n = 22)	Moderate GD (n = 64)	Severe GD (n = 77)
Age M (SD)	35.2 (9.6)	35.1 (10.8)	35.6 (9.3)	34.8 (9.6)
Gender %				
Women	25.2	22.7	28.1	23.4
Men	74.8	77.3	71.9	76.6
Education %				
Less than high school	19.1	19.0	14.5	23.0
High school	55.4	47.6	58.1	55.4
Occupational training	3.2	9.5	3.2	1.4
University	22.3	23.8	24.2	20.3
Occupational status %				
Working	72.0	95.5	77.4	61.0
Studying	2.5	-	3.2	2.6
Sick-leave	14.9	-	12.9	20.8
Unemployed	6.8	4.5	3.2	10.4
Other	3.7	-	3.2	5.2
Living Situation %				
Alone	30.6	27.3	29.0	32.9
With partner	20.0	31.8	16.1	19.7
With parents/friend	13.8	13.6	9.7	17.1
Single parent	9.4	4.5	11.3	9.2
With partner and children	25.6	22.7	32.3	21.1
Treatment center	0.6	-	1.6	-
Tobacco use %				
Yes	66.5	63.6	59.7	73.2
No	33.5	36.4	40.3	26.8
AUDIT M (SD)*	6.3 (6.0)	4.4 (3.6)	4.7 (3.5)	8.1 (7.5)
Harmful alcohol use %				
Yes	31.6	15.0	22.6	43.8
No	68.4	85.0	77.4	56.2
DUDIT M (SD)*	2.7 (7.2)	0.6 (1.7)	1.6 (5.5)	4.2 (8.9)
Problematic drug use %				
Yes	19.6	10.0	14.8	26.4
No	80.4	90.0	85.2	73.6

*Notes*. Severity of gambling disorder is defined by criteria fulfilled on the SCI-GD criterion A. 4–5 = mild, 6–7 = moderate, ≥ 8 = severe. AUDIT scores ≥ 6 for women and ≥ 8 for men indicate potential harmful alcohol consumption. DUDIT scores ≥ 1 for women and ≥ 3 for men indicate problematic drug use

*A significant linear trend was found with increasing GD severity, p < .05

There were no differences between participants with different severities of gambling disorder regarding gender (p = .831), education (p = .316), occupational status (p = .086), or living situation (p = .678), and no significant linear trend between age and gambling severity (p = .913). Most of the participants were tobacco users, 66.5%, and there was no difference in use between participants with different GD severities (p = .192).

The frequency of harmful alcohol use in the total sample as defined by AUDIT was 31.6% scores (≥ 6 for women and ≥ 8 for men indicate potential harmful alcohol consumption, see Bergman & Källmén, 2002). The frequencies based on gambling severity were 15% for mild GD, 22.6% for moderate GD and 43.8% for severe GD. There was a significant linear trend of increasing alcohol problems measured by AUDIT scores with increased GD severity (n = 155, Jonckheere-Terpstra test = 4365.0, p < .05). Pairwise comparisons using Mann-Whitney tests showed there was a significant difference between moderate (n = 64, M = 4.7, SD = 3.5) and severe (n = 77, M = 8.1, SD = 7.5) GD, p < .05, 95% CI for mean difference [1.5–5.4].

The frequency of illicit drug problems in the total sample as defined by DUDIT scores was 19.6% (≥ 1 for women and ≥ 3 for men indicate problematic drug use, see Berman et al., 2005). The frequencies based on gambling severity were 10% for mild GD, 14.8% for moderate GD and 26.4% for severe GD. There was a significant linear trend of increasing illicit drug problems measured by DUDIT scores (see Table 1) with increased GD severity (n = 153, Jonckheere-Terpstra test = 3943.5, p < .05). No significant differences between any of the specific severity groups were found when doing pairwise comparisons.

### Gambling Behaviors

In Table 2, frequencies, means and standard deviations of gambling behavior variables in the total sample and categorized by gambling severity are presented. In the total sample (n = 163) most had some form of online betting as their dominant gambling type, with 63.2% stating online casinos and 26.4% online sports betting as a dominant type. Only 6.7% stated betting in physical stores as a dominant type, while 4.9% was day trading on the stock market. There were no differences in frequencies of any of the dominant gambling types between the three different severity groups.

**Table 2:** *Gambling behaviors for total sample, mild, moderately and severely disordered gamblers. Data are presented as means and standard deviations M (SD) and in percent (%)*

	Total sample (n = 163)	Mild GD (n = 22)	Moderate GD (n = 64)	Severe GD (n = 77)
Dominant gambling type %				
Online slots/casino Online sports betting Physical store Daytrading Other	63.2 26.4 6.7 4.9 8.0	77.3 18.2 9.1 - 9.1	59.4 28.1 3.1 9.4 9.4	62.3 27.3 9.1 2.6 6.5
Reasons for gambling %				
Economic	60.1	59.1	70.3	51.9
Escape** Excitement Habit Self-harm	57.1 39.3 9.2 5.5	31.8 31.8 9.1 -	37.5 34.4 14.1 3.1	80.5 45.5 5.2 9.1
Age of gambling debut M (SD)*	20.4 (9.9)	23.7 (11.7)	20.4 (9.2)	19.4 (10.0)
Duration of problem M (SD)	7.5 (7.0)	5.6 (4.8)	7.1 (7.1)	8.3 (7.4)
NODS M (SD)**	6.3 (2.9)	4.3 (2.4)	5.8 (2.8)	7.2 (2.7)

*Notes*. Severity of gambling disorder is defined by criteria fulfilled on the SCI-GD criterion A. 4–5 = mild, 6–7 = moderate, ≥ 8 = severe. Total percentages exceed 100% for dominant gambling type and reasons for gambling as participants could give several answers

*A significant linear trend was found with increasing GD severity, p < .05

**A significant linear trend was found with increasing GD severity, p < .001

Several different reasons were given for gambling. In the total sample 60.1% reported gambling for economic reasons, 57.1% reported it as a form of escape, 39.3% due to excitement, 9.2% did it out of habit and 5.5% as a form of self-harm.

A Mantel-Haenszel test showed that there was a significant linear trend regarding using gambling as a form of escape with increasing severity (p < .001) with 80.5% of severely gambling disordered participants giving escape as a reason to gamble. For mild GD the corresponding frequency was 31.8% and for moderate GD 37.5%. The Odds Ratio (OR) when comparing the likelihood of stating escape as a reason between participants with severe GD and moderate GD were 6.89, meaning those with severe GD were 6.89 times more likely to state this reason compared to those with moderate GD. Between moderate and mild GD the OR was 1.29. No other reasons differed significantly between GD severities.

The participants in the total sample had an average age of gambling debut of 20.4 (SD = 9.9) years. A Jonckheere-Terpstra test revealed a significant linear trend of younger age of gambling debut with increased GD severity (n = 161, Jonckheere-Terpstra test = 3297.5, p < .05). Pairwise comparisons using Mann-Whitney tests revealed a significant difference between Mild (n = 22, M = 23.7, SD = 11.7) and severe (n = 76, M = 19.4, SD = 10.0) GD, p < .05, 95% CI for mean difference [-0.7–9.3]. Gambling had been problematic for participants for an average of 7.5 (SD = 7.0) years. There was no significant linear trend between the duration of gambling problems and GD severity (n = 162, Jonckheere-Terpstra test = 4386.5, p = .187).

Finally, the mean NODS score in the total sample was M = 6.3. There was a significant linear trend of higher NODS scores with increased GD severity (n = 158, Jonckheere-Terpstra test = 5291.0, p < .001). Pairwise comparisons using Mann-Whitney tests showed significant differences between all GD severities, with higher NODS scores corresponding to more severe GD. Mild (n = 22, M = 4.3, SD = 2.4) to moderate GD (n = 64, M = 5.8, SD = 2.8), p < .05, 95% CI for mean difference [0.1–2.9]. Moderate to severe GD (n = 77, M = 7.2, SD = 2.7), p < .001, 95% CI for mean difference [0.5–2.3]. Mild to severe GD, p < .001, 95% CI for mean difference [1.6–4.2].

### Depression, Anxiety, Emotion Regulation, Cognitive Distortions and Quality of life

In Table 3, adjusted means, unadjusted means and confidence intervals for depression, anxiety, emotional dysregulation, cognitive distortions and quality of life in all severity groups are presented, together with p-values.

**Table 3:** *ANCOVA results between different gambling severities on depression (PHQ-9), anxiety (GAD-7), emotional instability (DERS), gambling related cognitive distortions, (GBQ), and quality of life (BBQ):p-values, means and adjusted means with 95% confidence intervals*

Measure		Mild GD	Moderate GD	Severe GD	p-value
	Unadjusted	9.3	13.4	16.7	
	95% CI	[6.9–11.8]	[11.6–15.2]	[15.4–18.1]	
PHQ-9					< 0.001
	Adjusted	10.0	13.6	16.5	
	95% CI	[7.1–12.8]	[12.0–15.2]	[15.1–18.0]	
	Unadjusted	7.0	10.5	13.3	
	95% CI	[4.8–9.1]	[9.1–11.9]	[12.1–14.6]	
GAD-7					< 0.001
	Adjusted	7.8	10.7	13.1	
	95% CI	[5.4–10.2]	[9.4–12.0]	[11.8–14.3]	
	Unadjusted	28.9	42.8	50.5	
	95% CI	[22.5–35.2]	[38.6–47.1]	[46.7–54.2]	
DERS					< 0.001
	Adjusted	30.8	43.1	49.7	
	95% CI	[23.6–38.1]	[39.0–47.2]	[45.9–53.4]	
	Unadjusted	65.2	69.8	74.3	
	95% CI	[57.3–73.1]	[64.8–74.7]	[68.7–79.9]	
GBQ					= 0.131
	Adjusted	66.7	70.4	73.8	
	95% CI	[56.3–77.0]	[64.8–76.0]	[68.6–79.0]	
	Unadjusted	45.6	41.1	31.6	
	95% CI	[36.2–55.0]	[35.2–47.0]	[27.3–35.8]	
BBQ					< 0.05
	Adjusted	42.8	40.1	32.2	
	95% CI	[33.7–52.0]	[34.9–45.3]	[27.5–36.9]	

*Notes*. Severity of gambling disorder is defined by criteria fulfilled on the SCI-GD criterion A. 4–5 = mild, 6–7 = moderate, ≥ 8 = severe

The level of depression in the total sample, as represented by the mean PHQ-9 score, was M = 14.4 (SD = 6.9). An ANCOVA revealed a significant difference in levels of depression between the three different severities of GD when gender, age and possible ADHD was controlled for, F (2, 154) = 7.20, p < .001. Pairwise comparisons conducted using the Tukey-Kramer test revealed a significant difference between mild and severe GD, p < .001, 95%, CI for mean difference [2.7–10.0], and also between moderate and severe GD, p < .05, 95% CI for mean difference [0.3–5.5] with severe GD being associated with more depressive symptoms.

The level of anxiety in the total sample as measured by GAD-7 was M = 11.4 (SD = 5.8). An ANCOVA revealed a significant difference in GAD-7 scores between severity groups when controlling for gender, age and possible ADHD, F (2, 151) = 8.27, p < .001. Pairwise comparisons revealed significant differences between mild and severe GD, p < .001, 95% CI for mean difference [2.0–8.6], and between moderate and severe GD, p < .05, 95% CI for mean difference [0.2–4.6]. Severe GD was associated with more anxiety.

For emotional dysregulation measured by DERS the total sample mean was M = 44.6 (SD = 17.5). There were significant differences between severity groups when controlling for gender, age and possible ADHD, F (2, 146) = 7.93, p < .001. In pairwise comparisons a difference was found between mild and moderate GD, p < .05, 95% CI for mean difference [2.3–22.2], and between mild and severe GD, p < .001, 95% CI for mean difference [8.9–28.7]. No difference was found between moderate and severe GD, p = .054. Mild GD was associated with less problems with emotion regulation.

The level of cognitive distortions related to gambling in the total sample was M = 71.3 (SD = 22.0). There was no significant difference between levels of GD severity when controlling for gender, age and possible ADHD, F (2, 148) = 1.73, p = .13.

The total sample mean quality of life-level as measured by the BBQ was M = 37.1 (SD = 21.2). The three GD severity groups differed significantly regarding their quality of life when controlling for gender, age and possible ADHD, F (2, 146) = 2.34, p < .05 with lower quality of life scores with higher levels of GD severity. There were, however, no significant differences between any of the severity groups when conducting pairwise comparisons.

## Discussion

In this study we explored the clinical features of mild, moderate, and severe treatment-seeking patients with GD in our outpatient Clinic for gambling addiction and screen health. We found that there were no differences between the different severities of GD on any of the sociodemographic characteristics: gender, age, education level, occupational status, living situation and tobacco use.

We also found that a higher severity of gambling disorder corresponded to more alcohol and drug problems. Tobacco use was endorsed by a majority of participants regardless of severity.

A great majority of the individuals with severe GD reported “escape” as a reason for gambling and were several times more likely to do so than both those with mild and moderate GD. Those with severe GD had more symptoms of depression and anxiety than those with mild and moderate GD when controlling for gender, age and possible ADHD. Patients with moderate and severe GD were more emotionally dysregulated than those with a mild disorder when controlling for gender, age and possible ADHD. We also saw that cognitive distortions related to gambling were equally common in all three severity groups. There was an overall difference between the severity groups regarding quality of life. We found that online casino was the most common dominant gambling type independently of GD severity. Finally, a higher gambling severity was found to correspond to an earlier age of gambling debut.

Regarding the sociodemographic characteristics we found that the most common characteristics was being male, being between 30 and 40 years of age and having a secondary education or less. The majority reported living together with someone (living with partner, living with partner and children, or living with parent or friend) and the majority also reported being employed (working or being on sick leave). In addition to these overall findings, we found no difference between these sociodemographic characteristics of gender, age, education level, occupational status and living situation with regard to the severity of GD. This could mean that GD of all severities can be present and equally common regardless of sociodemographic status in a treatment seeking population. To our knowledge only one previous study reported sociodemographic differences between the GD severity levels specified in the DSM-5 and found that the mild GD group was younger and had a higher proportion of males compared to the moderate and severe group (Grant et al., 2017). With the Grant et al., (2017) study and our study, which is an exploratory study, it is hard to draw any final conclusions regarding the sociodemographic characteristics of GD severities. However. previous literature has reported similar sociodemographic characteristics in individuals with gambling problems. A recent review on demographic factors in problem online gamblers described that the most common characteristics were being male, single, being between 30 and 40 years old and having a secondary education (Mora-Salgueiro et al., 2021). Similarly, in 680 treatment-seeking pathological gamblers, variables such as being male, single or divorced, unemployed, or having a lower level of education was found (Pavarin et al., 2018). The population in our study differed from the previous studies regarding occupational status and living situation (i.e. they were more often employed and more often living together with someone). However, demographic factors vary a great deal between different studies (Mora-Salgueiro et al., 2021) therefore it is not possible to conclude that our population is markedly different from other populations with problematic gambling.

Regarding the concomitant intake of alcohol and illicit drugs, we found that a higher severity of gambling problems was associated with increased alcohol problems. In the severe group 43.8% had a problematic alcohol use, defined by a score of 6 > for women and 8 > for men, compared to the moderate group in where 22.6% and the mild group where 15% had a problematic use. For clinicians this is noteworthy. As it is known that comorbid alcohol and substance problems are common among problem and pathological gamblers (Lorrains et al., 2011; Mora-Salgueiro et al., 2021) clinicians should always screen for this when treating gambling addicted patients. However, it should be increasingly important to assess and treat potential alcohol problems the more severe a gambling disorder is, as a lower use of alcohol has been found to be a likely predictor for treatment success in patients with GD (Merkouris et al., 2016).

Our findings also show that within the group of pathological gamblers, a more severe GD is associated with increased problems with other substances. In the severe group 26.4% had a problematic drug use compared to the moderate group in where 14.8% and in the mild group 10% had a problematic use. Drug use has not been found to have a similar predictive effect on treatment success as problematic alcohol use (Merkouris et al., 2016). Even so, illicit drug use should also be important to assess and treat in its own right. It should be noted that though a more severe GD is associated with more problems with other substances, harmful alcohol and illicit drug habits were present in all groups.

The association between gambling disorder and other addictions also strengthen the reclassification of GD in the DSM-5 as an addictive disorder. In contrast to our findings, Grant et al., (2017) did not find any differences between categorical alcohol- or substance use disorders between any of the severity groups. This difference may largely be due to differences in how alcohol, and substance use problems were measured and analyzed. Instead of using categorical disorders we measured degree of problems with the AUDIT and DUDIT questionnaires. Measuring degree of problems instead of categorical disorders is another way to describe the groups, and it can be a way to find clinically meaningful differences that aren’t always apparent when comparing diagnoses. Indeed, individuals fulfilling criteria for a disorder can still differ in their severity and the degree of problems the disorder causes. Even so, the only difference we found between groups in pairwise comparisons was that the severe GD group had more alcohol problems compared to those with moderate GD. However, there was still a meaningful association between alcohol and drug problems and GD severity represented by significant linear trends between these.

It is well established that a higher rate of gambling disorder is associated with a greater substance use. Prior research from a clinical perspective has showed a very similar picture between GD and substance abuse, such as initial excitement and reward from the behavior to a loss of control despite negative consequences (for review see Grant & Chamberlain, 2020). Further a shared genetic predisposal to both gambling and alcohol problems have been seen in twin studies (Potenza, 2017). In light of this, it is not unreasonable that a more severe gambling disorder is also associated to more problems with alcohol and other substances.

We also found that a majority of participants endorsed tobacco use. Unlike the Grant et al., (2017) study where more nicotine use was seen in the severe group compared to the mild GD group, we did not find a difference in tobacco use between the severity groups in our study (mild 63.6%; moderate 59.7%; severe group 73.2%). Even though it is well known that problem and pathological gambling is highly comorbid with a nicotine dependency with prevalence rates as high as 40–60% (Lorrains et al., 2011; Dowling et al., 2015a) little attention has been paid to this comorbidity in the research literature. This is noteworthy as the commonness of this comorbidity means gambling addicted individuals are likely disproportionately affected by tobacco related health problems and death. Indeed, in a review of global statistics on alcohol, tobacco and illicit drugs, tobacco was found to have the highest substance-attributable mortality rate (100.7 deaths per 100 000 people) and disability-adjusted-life-years reduction of all substances (Peacock et al., 2018). Apart from the apparent harmful health consequences of nicotine use, smoking problem gamblers has also been seen having a more severe gambling pathology with a higher rate of other comorbidities such as substance use disorders and anxiety (Petry & Oncken, 2002; Grant et al., 2008) and they also experience a stronger urge to gamble (Grant & Potenza, 2005). It has also been seen that gamblers who smoke tend to bet larger sums and spend more time with activities connected to gambling (Petry & Oncken, 2002). They also have greater financial problems (Potenza et al., 2004). It is thus clear that concomitant tobacco use is indicative of more gambling related problems, even though there was no clear effect regarding GD severity in our study. With the harmful effects of tobacco use in mind, and its high frequency among patients with GD, screening for and offering help to end a nicotine dependency at facilities offering treatment for gambling problems might be a promising way to improve health outcomes for this patient group.

The likeliness to state “escape” as a reason for gambling increased with increasing GD severity. As many as 80.5% of those with a severe disorder stated this reason compared to 37.5% for moderate and 31.8% for mild GD. We believe this is a highly interesting finding and potentially interesting for clinicians when planning and executing treatment for their patients with severe GD. The statement “escape” somewhat corresponds to one of the criteria from the DSM-5 (APA, 2013), namely “often gambles when feeling distressed”. Interestingly this criterion has not been found to discriminate well between different severities of GD in earlier studies (Toce-Gerstein et al., 2003; Sleczka et al., 2015; Chamberlain et al., 2017). A possible explanation for this might be that gamblers definition of “escape” is broader than the avoidance of distressing thoughts and feelings.

Further, gambling for escape can also be a way to handle a depressive state. In an online survey study on 282 recreational gamblers that assessed depression severity, expectancies of escape, excitement and problem gambling it was found that gambling to escape moderated the relationship between depression and problem gambling. This could indicate that individuals using gambling as a way to escape a depressive state are more at risk of developing gambling problems (Vaughan & Flack, 2021).

It is interesting then, that while all severity groups showed elevated levels of depression and anxiety which is in line with previous research showing high rates of comorbid mood disorders (23.1%) and comorbid anxiety disorders (17.6%) among treatment-seeking pathological gamblers (Dowling et al., 2015a), our severely disordered gamblers had more symptoms of depression and anxiety than those with mild and moderate GD when we controlled for gender, age and possible ADHD. This result is somewhat in line with the study by Grant et al., (2017) that also found a difference between severity groups on state anxiety and depression scores. In contrast however, they instead found moderate and severe GD to be similar, with mild GD having lower rates of depression and anxiety.

In Nower et al., (2021) revised pathways model of gambling, a distinct subtype of problem gamblers, emotionally vulnerable gamblers (pathway 2), are described. These gamblers have a higher extent of psychosocial risk factors such as problems with depression and anxiety and experiences of childhood maltreatment. These risk factors are thought to be present before a gambling problem sets in, and gambling is first used in order to relieve aversive affective states by means of escape or arousal. It is possible that this subgroup of problem gamblers is overrepresented among individuals with severe GD, explaining the high endorsement of “escape” as a reason for gambling, as well as the higher rates of depression and anxiety in this group. We can also speculate in that our severe GD patients reaches a state labeled “dark flow” by researchers (for review see Schluter & Hodgins, 2019). Gamblers refer to a trance-like state of absorption and occupation by the game in where potentially negative consequences for the player may happen in where they spend more money than planned. It has been found that “dark flow” is positively correlated to positive affect during play and that dark flow and depression predicts gambling problems (Dixon et al., 2019). Perhaps the dark flow is a state that the severe gamblers want to escape to.

Those with a moderate or severe GD had more problems with emotion regulation than those with a mild GD. Emotion (dys)regulation has been argued to play an important role in the development and maintenance of GD (Rogier & Velotti, 2018) and earlier studies have indeed shown problems with emotion regulation to be associated with GD (for a review and meta-analysis see Velotti et al., 2021). Our results are in line with this, as those with a more severe GD also have more problems with emotion regulation. It has been recognized that individuals with ADHD also have problems with emotion regulation. In a study of 98 patients with GD it was found that those that also had ADHD had more problems with emotion regulation than those without ADHD (Mestre-Bach et al., 2021). They also found that difficulties in emotion regulation mediated the relationship between ADHD and gambling severity. It is interesting then that we found that patients with moderate and severe GD had more difficulties with emotion regulation than those with mild GD, even when controlling for possible ADHD. It is likely that difficulties with emotion regulation are a risk factor for developing a more severe disorder, and that ADHD is only one of several possible reasons for such difficulties. Again, this finding is in line with the pathways model (Nower et al., 2021) as the “emotionally vulnerable gamblers” is the subtype with the most severe gambling problems in the model.

We found that cognitive distortions related to gambling (i.e., a belief that luck is dispositional, an illusion of control over random events) were as common in all severity groups. Previous research has identified a number of known cognitive distortions related to gambling and these have been implicated in the development and maintenance of gambling problems (Fortune & Goodie, 2012). In longitudinal studies there is a known association that a higher rate of these cognitive distortions leads to more severe gambling problems later in life (Leonard & Williams, 2016; Nicholson et al., 2016). Still, we found no difference between the different severities of GD on these distortions. Hypothetically, this could be explained by a lessening of the association between cognitive distortions and gambling severity when the gambling problem has reached the level of disorder. It might be that further increases in severity beyond the level of disorder is influenced more by other factors. In a study of 177 treatment-seeking problem gamblers in Hong Kong, Wong et al., (2018) found that while gambling related cognitive distortions were associated with higher gambling severity, negative psychological states significantly moderated the relationship between these distortions and severity. This shows that cognitive distortions in themselves might not always lead to more severe gambling problems, and that they pose a greater problem when paired with negative mood states. This is supported by the fact that cognitive distortions related to gambling can be found among both non-problem and problem gamblers, and that there is a clear heterogeneity between individuals in each group (Leonard & Williams, 2016). It might be then, that the differences in severity on the level of disorder is affected more by emotional, rather than cognitive variables.

All severity groups were found to have low quality of life scores. A score < 52 on the BBQ scale is considered a clinical level of life dissatisfaction (Lindner, 2016). All BBQ scores were well below this level (mild GD 42.8, moderate GD 40.1, severe GD 32.2). This was expected as GD is known to be associated with poor quality of life (for a review see Potenza et al., 2019). Our study indicates that this is true for GD of any severity. There was also an overall difference between severities regarding quality of life, although no specific differences were found in pairwise comparisons. This result is however still interesting as a lower quality of life after treatment can be related to relapse in GD (Sander & Peters, 2009). This means that severity of gambling could potentially affect risk for relapse after treatment indirectly via an individual’s quality of life. So far, the severity rates potential for predicting relapse has only been examined in one study, which specifically examined relapse during treatment only. Here, severity was not found to predict relapse during the course of treatment (Mestre-Bach et al., 2019).

In our participants, the most common dominant gambling type was online casino, with 63.2%, followed by online sports betting at 26.4%. This comes as no surprise as gambling problems are more common among gamblers partaking in casino gambling, bingo and sports betting (Mazar et al., 2020). It also seems like gambling online is associated with more severe gambling problems. In a recent study by Wall et al. (2021) who investigated 7463 problem gamblers calling a helpline in Sweden, it was found that gambling severity was associated with gambling involvement and that the strength of association varied by game type. Online gambling was associated with the highest problem gambling severity. Interestingly, online gamblers have also been found to be less likely to seek treatment (Gainsbury, 2015; Hing et al., 2015). Keeping this in mind, the true prevalence of these gambling types among gambling disordered individuals might be even higher than in our study, as a lower likelihood to seek treatment among these gamblers could present a selection bias. Even though online gambling is associated with more severe gambling problems, we did not find a difference in dominant gambling type among the different levels of GD. Even at a mild level of disorder, online casinos and/or online sports betting was the dominant gambling type for most of the participants. It might be that when gambling problems approach the level of disorder, even when in a mild form, these types of games are already dominant. This notion is supported by a study by Håkansson et al., (2017) in which an overwhelming majority of 84% of Swedish GD patients seeking treatment reported online casino or online sports betting as one of their problem games.

We also found an association between earlier age of gambling debut and more severe GD. In pairwise comparisons we found a specific difference between the mild and severe groups, with those with severe GD showing an earlier gambling debut. In contrast, Grant et al., (2017) found no differences between any of the severities. Also, in Grant et al., (2017) there seems to be no trend of decreasing raw scores with increasing severity. Due to these conflicting results, it is so far too early to tell whether an earlier gambling debut is in fact indicative of a more severe disorder later in life. Our finding is however interesting, in light of alcohol use disorder, as there is a known association between early onset of drinking and heavy drinking and alcohol related problems later in life (Grant & Dawson, 1997; Hingson et al., 2006).

So far, only a few studies have explored if the severity levels specified in the DSM-5 translate to meaningful clinical differences among treatment-seeking gamblers. Grant et al., (2017) found that those with mild GD differed from moderate and severe GD regarding age of gambling debut, money lost due to gambling, nicotine consumption, and measures of anxiety, depressive symptoms, and quality of life. However, no difference was found between moderate and severe GD on these measures. In addition, they found no differences whatsoever regarding categorical mood, anxiety or substance use disorders. Based on this they argue that the DSM-5 severity levels might lack clinical utility, as there weren’t clear differences between each severity level. The same is argued by Mestre-Bach et al., (2019), as they didn’t find the DSM-5 severity levels to have any predictive capacity regarding treatment outcome. Indeed, a simple symptom count might be too simplistic to measure syndrome severity as there is evidence indicating that some specific criteria (i.e. needing economical bailouts) are more commonly associated with more severe pathology (Toce-Gerstein et al., 2003; Sleczka et al., 2015; Chamberlain et al., 2017). However, there is also some evidence for the merit of a symptom count. Number of criteria fulfilled has been found to be positively associated to symptoms of depression as well as to gambling related cognitive distortions (Jiménez-Murcia et al., 2020), both meaningful clinical variables. In contrast to the study by Grant et al., (2017) we found the severe group to in several cases differ from the other severity levels. We would argue that, in line with our hypothesis, the increased levels of anxiety and depression, and the increased tendency to gamble to escape in those with severe GD is indeed clinically meaningful, as is the increased level of alcohol- and drug-problems associated with increased GD severity. This lends merit to the severity levels having clinical relevance. It must also be taken into account that such a classification is easy to use for clinicians, as it is a straightforward approach.

However, one must be aware that there are still a low number of studies on this subject, and the results so far are somewhat conflicting. Therefore, there is in our assessment still too little evidence to make a final verdict on the usefulness of the DSM-5 severity levels. This is interesting as DSM-5 criteria are often used to make clinical decisions. Indeed, locally in the region in Sweden where our research is based, DSM-5 severity levels are used to decide whether patients should get specialized treatment or not.

This study presents several strengths and limitations. The strengths are that all participants were recruited from a real treatment seeking population in ordinary care, and thus represent a treatment-seeking gambler population. Participants were reliably diagnosed in their gambling disorder using gold-standard clinical interviews (SCI-GD). In addition to this, self-report questionnaires were used to screen for a large number of clinically relevant variables. All measures were taken during or around the first visit to the clinic before participants were enrolled in treatment. This is a strength as we can be sure that the variables haven’t been affected by treatment. The limitations are that the study had only n = 163 participants. In addition to this, the sample size was based on convenience and not on a predetermined power analysis. As the study was conducted in a clinical setting there was a limitation in recruitment based on the number of patients seeking treatment at the clinic, and we chose to end recruitment after two years. A better powered study would have reduced the risk of type II error and might have been able to reveal clearer differences between the severity levels. Another limitation was that our sample was from a treatment-seeking population. In this study we were mainly interested in gamblers encountered in the clinic, however this still means that our results cannot be generalized to everyone with a gambling disorder. Finally, all participants were of Swedish origin and of an unknown ethnic composition. It is known that the prevalence of problem gambling can differ between different countries and cultures (Williams et al., 2012) and between ethnic groups (for example, see Forrest & Wardle 2011; Caler et al., 2017). It is possible that different gambling habits also means that the severity of GD is expressed differently within different cultures or ethnic groups. It is therefore not possible to generalize our results to other groups.

Several areas are of interest for future research. Since GD is included among the substance use disorders in the DSM, an important next step may be to better calibrate the diagnoses so that they are all on the same metric of severity. Due to somewhat conflicting results in the literature, it is still an open question whether the DSM-5 severity levels represent a clinically meaningful way of categorizing GD severity, and if so, what clinical characteristics are typical for the different levels of severity. Thus, it would be valuable with more studies exploring the different GD severities to paint a clearer picture of possible meaningful clinical differences between these. Such studies should aim to test specific hypotheses generated by earlier studies such as this one. Another interesting area of research could be to further examine if any particular pathway to gambling (Nower et al., 2021) is overrepresented in any of the severity groups. More knowledge of this could help clinicians better tailor their interventions depending on the level of disorder.

In sum, we found that a more severe GD was associated with increased problems with alcohol and illicit drugs, patients with severe GD were more depressed and anxious, and more often gambled to escape than those with mild and moderate GD. Gamblers with moderate and severe GD had more problems with emotional regulation. GD regardless of severity was associated with low quality of life. It seems then, that relevant clinical variables such as problems with depression and anxiety and reasons for gambling differ between severity levels, and this is important to keep track of when meeting patients with GD in the clinic. Patients with GD are often treated in clinics specialized in gambling but not seldom also treated within the community health care or in support groups. There is always a risk that the treatment only focuses on gambling alone unaccompanied by a treatment plan for the psychiatric comorbidity. Our findings emphasize the importance of screening patients with severe GD for alcohol and drug use, anxiety, depression, and emotional dysregulation.

## Data Availability

Data will be made available upon request.

## Code Availability

n/a.
